# A spatiotemporal natural-human database to evaluate road development impacts in an Amazon trinational frontier

**DOI:** 10.1038/s41597-019-0093-7

**Published:** 2019-06-17

**Authors:** Geraldine Klarenberg, Rafael Muñoz-Carpena, Stephen Perz, Christopher Baraloto, Matthew Marsik, Jane Southworth, Likai Zhu

**Affiliations:** 1 Department of Wildlife Ecology and Conservation, University of Florida, Gainesville, Florida USA; 2 Department of Agricultural and Biological Engineering, University of Florida, Gainesville, Florida USA; 3 Department of Sociology and Criminology & Law, University of Florida, Gainesville, Florida USA; 4 International Center for Tropical Botany, Department of Biological Sciences, Florida International University, Miami, Florida USA; 5 Integrated Data Repository, Clinical and Translational Science Institute and UF Health, University of Florida, Gainesville, Florida, USA; 6 Department of Geography, University of Florida, Gainesville, Florida, USA; 7 Shandong Provincial Key Laboratory of Water and Soil Conservation & Environmental Protection, College of Resources and Environment, Linyi University, Linyi, China

**Keywords:** Biodiversity, Environmental impact, Tropical ecology, Interdisciplinary studies, Environmental impact

## Abstract

Road construction and paving bring socio-economic benefits to receiving regions but can also be drivers of deforestation and land cover change. Road infrastructure often increases migration and illegal economic activities, which in turn affect local hydrology, wildlife, vegetation structure and dynamics, and biodiversity. To evaluate the full breadth of impacts from a coupled natural-human systems perspective, information is needed over a sufficient timespan to include pre- and post-road paving conditions. In addition, the spatial scale should be appropriate to link local human activities and biophysical system components, while also allowing for upscaling to the regional scale. A database was developed for the tri-national frontier in the Southwestern Amazon, where the Inter-Oceanic Highway was constructed through an area of high biological value and cultural diversity. Extensive socio-economic surveys and botanical field work are combined with remote sensing and reanalysis data to provide a rich and unique database, suitable for coupled natural-human systems research.

## Abstract (summary)

**Table:** 

Design Type(s)	data integration objective • biodiversity assessment objective • stimulus or stress design
Measurement Type(s)	Homo sapiens • Biophysical Process • snapshot data
Technology Type(s)	survey method • digital curation
Factor Type(s)	Resolution • geographic location • temporal_interval
Sample Characteristic(s)	Peru • forest ecosystem • Brazil • Bolivia

Machine-accessible metadata file describing the reported data (ISA-Tab format)

## Background & Summary

Road infrastructure development is on the rise, and is predicted to increase 60% in total length by 2050, compared to 2010^1^. From a macro-economic perspective, road infrastructure is described as a necessity, as the increase of trade and economic growth will require gateways and corridor infrastructure for resource extraction, transport, processing and export^2^. Having quality infrastructure is considered the key to competitiveness on a global level because it integrates national markets and provides access to international markets. At a regional level, population growth and the increasing proportion of people living in cities represent a driver of increased mobility and thus increase the need for infrastructure. However, the addition of new infrastructure is projected to take place predominantly in emerging economies^3^; as a result, many of these roads will be constructed in wilderness and pristine areas^4^. Negative ecological impacts of roads from increased anthropogenic disturbance on forest ecosystems have been recognized in studies that have documented deforestation, increased logging, increased fire, loss of biodiversity, decreased mobility and increased mortality of wildlife, and hydrological changes^4–12^. Previous research predicts road impacts to be highest in areas high in biodiversity and carbon storage^1^, i.e. tropical forest regions.

The Amazon forest provides a number of locally, regionally and globally important ecosystem services^9,13^. The Amazon contains multiple biodiversity hotspots^14,15^, provides timber and non-timber forest products (NTFPs), and is home to dozens of distinct indigenous cultures. At the global level, carbon storage and nutrient cycling are important supporting ecosystem services, as well as hydrological regulation^5^ and climate regulation through freshwater discharge and vegetation-atmosphere feedbacks^16,17^. Roughly 53% of the total area of the Amazon is at risk from current or near-term threats including transportation, mining, agriculture, and logging^18^. This is a very conservative finding, since it does not include the loss of forest associated with road infrastructure, or the increased access to the forest interior due to road connectivity^18^. Numerous studies raise concerns about the Amazon reaching a tipping point, believed to be around 40% deforestation^17,19–23^ and this tipping point can be accelerated by recent changes in global climate^24^. Many of these studies predict a shift in states from a system dominated by tropical forest to a savanna-dominated system, or large-scale rainforest die-back.

As climate variability and human disturbances have become major sources of concern^16,25^, there is an increasing need to conduct long-term studies that consider road development as part of a complex coupled human and natural (CNH) system with specific spatio-temporal characteristics. Particularly in tropical forest areas where livelihoods often depend on natural resource management, and where ecosystem services have global importance, the integration of data across scales in social and ecological sub-systems becomes vitally important. With multi-temporal, cross-scale social and ecological data, we can conduct research that adopts a comprehensive view of the system to evaluate the advantages and disadvantages of road development.

With these priorities in mind, we collected and compiled data for a portion of the Amazon now being impacted by road development. The area in question is a tri-national frontier area where the states of Madre de Dios (Peru), Acre (Brazil) and Pando (Bolivia) meet, also known as the MAP region. The Inter-Oceanic Highway (IOH), which connects Pacific ports in Peru to Atlantic ports in Brazil, was constructed through the MAP area between the 1980s and 2011, with increased construction activity from 2002 onwards. The study region is a global hotspot of biodiversity, and historically has had a resource-dependent economy. It is also located in relatively remote portions of each of the three countries represented. The process of economic integration of the area through highway paving is therefore of great interest, and the fact that there are three countries involved, with different socio-economic circumstances and environmental and social policies, makes this area of interest from a comparative point of view. The MAP frontier has been described in detail in previous studies^10,11,26–34^. Our database is unique as it covers time periods before, during and after highway construction, and contains data pertaining to both the social and ecological sub-systems. In spatial terms, a large part of the data is available for 100 resource-dependent communities, as opposed to municipal or state units, offering a more refined and livelihoods-focused set of information. Certain variables are not available at the local or state level, but only at the country level; this is due to the disparities in data availability between states in different countries. Our focus in including governmental data was on compiling data uniformly available across the tri-national frontier area, and covering the time period before, during and after highway construction. These criteria constrained variable selection as certain variables had gaps in time series, or missing data for certain countries. It must be stressed that there are definitely more data available for certain countries and time periods, but that this dataset contains the data that are uniformly available across time and space. Recommendations further on, in the Usage Notes, highlight other data that could be combined with this dataset.

Various components of this database have been used in studies that assessed the human sub-system^11,35^, the biophysical sub-system^36,37^, or the interactions between the two^26,28,34,38^. We anticipate that these data can be of broader interest for a wide range of future studies, particularly for the evaluation of coupled human and natural systems theories, simulation and dynamic models, as well as research focusing on improving environmental impact assessments conducted for road development projects.

## Methods

### Data collection and compilation

**Fig. 1:**
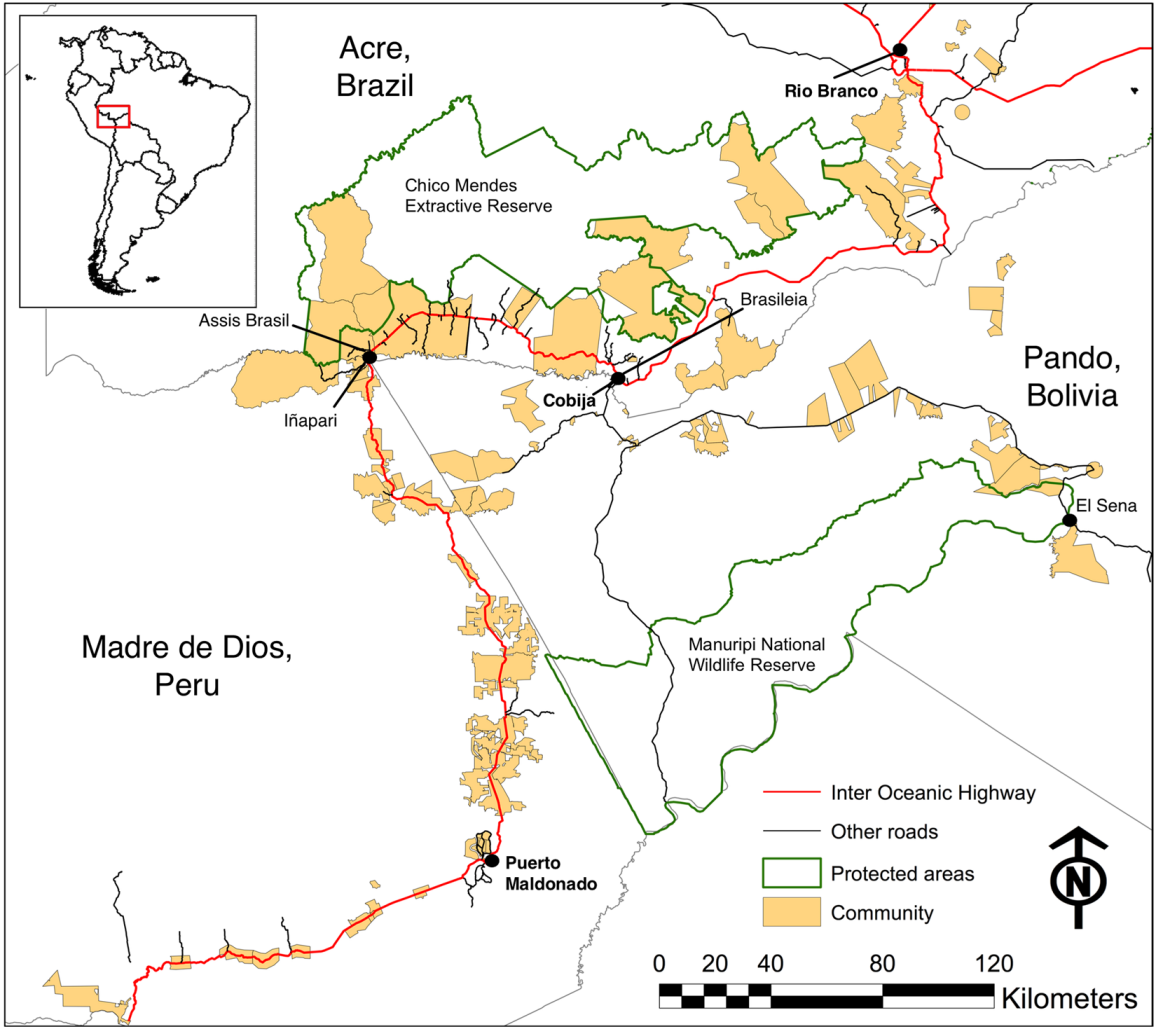
The MAP area - Madre de Dios (Peru), Acre (Brazil) and Pando (Bolivia) - with 100 communities, the Inter-Oceanic Highway (IOH), other roads and protected areas.

The database contains information on infrastructure, social and ecological sub-systems in time series (“dynamic variables”, Online-only Table 1), with variables available at the global, country, community and point levels. A “community” is defined as a distinct rural land tenure unit and/or population center, as employed in field surveys in the MAP area^28^, Fig. 1. Data compilation was primarily focused on the time period 1980–2010 (the road construction period), although data availability over that period varies (Online-only Tables 1 and 2). This database is a combination of information obtained from field work in communities in the study area, reanalysis data from larger, often global, datasets, and information derived from remote sensing. Secondary data sources were accessed during 2013–2015. There are also data records included in the database that are not dynamic and provide stationary spatial and qualitative information; a number of these are depicted in Fig. 1, and Online-only Table 2.

### Human variables

The field surveys involved collection of data from 100 human communities across the MAP frontier as detailed in previous publications^26,28^. The surveys were implemented in two phases, focusing first on communities, and then on households within these communities. Community boundaries were identified in a Geographic Information System (GIS) with the help of administrative data sources such as cadastral maps and zoning plans^30^. The focus was on communities around the IOH in Madre de Dios (Peru) and Acre (Brazil) and near major roads in Pando (Bolivia) along a distance gradient from the IOH. Systematic geographic sampling along primary roads such as the IOH led to a selection of communities with varying distances to regional capitals (and to the IOH in Pando) and more or less regular distances along roads, Fig. 1. Those then served as a sampling frame for selection of fewer communities for more in-depth fieldwork at the household level in the second phase.

In 2007 and 2008, faculty and students from the University of Florida (UF), the National Amazonian University of Madre de Dios (UNAMAD), the Amazonian University of Pando (UAP) and the Federal University of Acre (UFAC) interviewed current and past community representatives using a structured questionnaire with open-ended questions. In Madre de Dios, 88 interviews were conducted in 41 communities, in Pando, 111 interviews came from 37 communities, and in Acre, 93 interviews resulted in 25 communities. Those interviewed were current or previous community representatives and long-time residents. This initially resulted in 292 interviews in 103 communities, but some communities were later dropped because they were deemed too urban, or combined because they were found to be part of the same community^28^. The questionnaire covered interviewee characteristics, general information about the community, community population, community infrastructure, governmental and non-governmental projects, community livelihood activities, change in the importance of those activities in the past 5 years, change in availability of forest resources in the past 5 years, marketing of key products, conflicts over natural resources, and community response to fires. GPS information was collected to calculate distances along roads to nearest markets and regional capitals. Information gathered on community population changes (as number of families) included the reported number of families in a community as of 2007 (averaged among leader estimates), families considered community members but living elsewhere (usually a local town), number of families joining the community in the last 5 years, and number of families leaving the community in the last 5 years. Based on this information, we calculated the number of families in 2002, the change in number of families from 2002 to 2007 and estimated the number of families in 2012 using the 2002–2007 exponential growth rate. Using rural growth rates for a number of periods pre-2002 (based on rural populations for the states in which the communities were located, as reported in several censuses), population growth rates were extrapolated back to 1987^27^. Using the land areas of the communities, family densities over time were calculated. Changes in urban population size in the state capitals and the nearest markets were obtained from census data over time for each of the three countries, which included migration estimates.

In 2008 and 2009, the second phase of the socio-economic system sampling took place. Household interviews were conducted in roughly a quarter of the communities based on their location, paving status and tenure types. These communities were known to be representative of the larger community sample in terms of the sampling criteria^11^. Random sampling of households within each community was pursued where possible, and information from the previous phase was used to determine sampling ratios; sampling has been detailed in a previous publication^29^. In summary, sampling of communities was done systematically along roads. At the household level, lists of households were obtained where available; but due to changes in community populations, such lists were not always accurate. The lists were updated with community leaders before sampling. Where lists were not available, sampling was done systematically along roads and paths and thus by spatial location within community boundaries. This made sampling in effect stratified, e.g. sampling along all roads within the community. We also based sampling on reported populations, whether in state data or from community leader reports; and sampled more heavily in small communities to manage sample sizes and thus sampling errors. In Pando, 164 households were interviewed in 8 communities in 2008 by UF and UAP faculty and student teams. In Madre de Dios, 12 communities were visited by UF and UNAMAD teams in the same year, resulting in 312 household interviews. In 2009, 266 household interviews were conducted in 7 distinct tenure areas in Acre by UF and UFAC teams. As these data are not available as time series (as “dynamic variables”), we describe the resulting variables under the section “Stationary variables”.

Road paving is expressed as a proportion between 0 (no paving) and 1 (fully paved) and refers to the extent of paving at a given point in time for the road segment between two towns in which a community is located. From field observations, key informants and official documents, we obtained information on when the IOH was paved to each town in the MAP frontier, and interpolated paving extent as a linear process between locations and time points. We imputed travel speeds along primary roads (highways, paved and unpaved) and secondary roads (unpaved) in order to calculate travel times. We then calculated travel times (in minutes) based on the type of roads and proportion paved for each year, in order to create time series of travel times from each community to its nearest town and regional capital. As paving progressed, travel times declined.

Information was collected on tenure rules and the (perceived) enforcement of these rules for each community. Tenure rules are based on official documents such as zoning plans which indicate access and use rules for different kinds of lands, notably any deforestation limits. Enforcement was based on key informant interviews, workshops with stakeholders, and news reports of state actions against rule infractions. We created time series for tenure rules on deforestation limits based on the timing of official designations of lands with specific deforestation rules, and used values from 0 to 1 to reflect permitted deforestation areas as proportions of landholdings by tenure type. The values between 0 and 1 represent 0 to 100% deforestation allowed. Enforcement values reflect probability of state action in response to infractions against use rules, based on perceptions by informants and stakeholders, and news reports. The values run from 0 to 1, with higher values indicating a higher probability of state responses against infractions.

The community and household studies were approved by the University of Florida Institutional Review Board 02 (http://irb.ufl.edu/irb02.html, ref: 2007-U-0049 and 2007-U-0294). All participants were presented with the purpose of the questionnaire, and informed consent was obtained from all participants verbally. There were no participants younger than 18 involved in the studies.

At the country level, a number of socio-economic indicators were collected from the World Bank Databank (http://databank.worldbank.org/data/home.aspx), at annual resolution. As state-level data were not available uniformly over space and time, country-level data were included that could be informative of higher-level socio-economic and environmental policies. We examined a number of variables from the World Bank database and selected those that could potentially be relevant to research on deforestation, highway paving and frontier expansion, such as Gross Domestic Product (GDP) and GDP associated with natural resources extraction, electricity consumption and generation (as this often also involves the use natural resources), agricultural land and protected areas. The World Bank database holds data on a large number of variables, but we selected only those that had continuous data for the time period in question. The human variables are: electricity from non-renewable resources, electricity from renewable sources (excluding hydroelectric), foreign direct investment, profit from forests, Gross Domestic Product (GDP), GDP growth, GDP growth per capita, profit from natural gas, minerals, crude oil and forests, total profit from natural resources, foreign direct investment, life expectancy at birth, power consumption, electricity production from renewable and from non-renewable resources.

### Biophysical variables

Monthly data sets were sourced from the Climatic Research Unit (CRU) at the University of East Anglia (https://crudata.uea.ac.uk/cru/data/hrg/) for the minimum, mean and maximum temperatures, as well as for precipitation and potential evapotranspiration. The mean, minimum and maximum temperatures (in °C) and precipitation (in mm) were obtained at a resolution of 0.5 × 0.5°^39^. We assumed that the grid value represented only the property of grid center, and then utilized ordinary Kriging interpolation method to downscale raw data into new data at a 1-km finer resolution. Generally, for each community one or more pixels were included, so we assigned the value of each community using an area-weighted average method given that some pixels around community boundaries were not completely within community area. Potential evapotranspiration (in mm) is also included in the CRU data set, and is calculated from a variant of the Penman-Monteith formula, using mean, minimum and maximum temperature, vapor pressure and cloud cover^39^.

Soil moisture (SM) data come from the National Oceanic and Atmospheric Administration (NOAA) Climate Prediction Center (CPC) model at a resolution of 0.5 × 0.5°, which uses CPC precipitation data and temperature data from the NCEP/NCAR Reanalysis^40^. These data are downloaded from the NOAA Earth System Research Laboratory (ESRL) website (https://www.esrl.noaa.gov/psd/data/gridded/data.cpcsoil.html). They are provided as average soil moisture in terms of water height equivalents (mm). As with the other data sets, the soil moisture data are calculated as an area-weighted average time series for each community polygon.

Inferred plant species richness (SR) is calculated for vegetation from Landsat imagery by applying a method that is based on the Shannon entropy of pixel intensity^41,42^, at annual time steps. Landsat imagery at 30 m resolution were collected for each year^42^, and the images were analysed for each visible band (blue, green and red). A wavelet-based texture retrieval method was used that applied wavelet analysis to decompose local (pixel) signals. The *α* diversity for vegetation is calculated as Shannon’s entropy index of the green band - the spectral heterogeneity measured by reflectance is used. Because different plant species have different reflectance levels in the visible bands^43^ at macroscale, the range of reflectance is indicative of tree diversity. If the range of reflectivity is higher, the entropy is higher, hence the plant diversity is higher. The entropy gradient is linearly proportional to the plant species richness gradient and thus provides plant diversity as a macro-ecological variable^42^; plant species richness per community was calculated in area-weighted manner. Since there is a degree of variation associated with this reflectance-based method, inferred plant species richness should be interpreted relatively, not in the absolute sense. The broad patterns that emerge are representative of the study area: high diversity and lower coefficients of variation for communities in Bolivia (mostly old growth forest with little to no highway paving), intermediate diversity and higher coefficients of variation in Peru (an area subject to naturally higher disturbance and turnover rates^44–46^), and lower diversity and sudden shifts in Brazil (areas with more anthropogenic influences and earliest highway paving).

The data on area covered by forest in each community (as a fraction) were generated with methods from a previous study in the Bolivian part of the MAP area^30,36,47^. The method for forest-nonforest (FNF) classification relied on Landsat images (4 and 5 TM and 7 ETM+) courtesy of the U.S. Geological Survey of the area from 1986, 1991, 1996, 2000, 2005 and 2010. Images were selected during the dry season, to minimize the impact of cloud cover and smoke. Corrections for atmospheric and seasonal differences were applied to the images, and all images were georeferenced to less than 15 m error, with the Maryland Global Land Cover Facility 2000 Geocover as base images. After mosaicking and removal of clouds, shadows and water with a Principal Component Analysis (PCA) image differencing and thresholding method, derived image products for bands 4, 5 and 7 were generated to assist in classification. These products were tasselled cap indices (producing three bands that represent brightness, greenness and wetness), a mid-infrared index and a 3 × 3 moving variance window calculation of each pixel. The latter gives a measure of texture, which is helpful in classifying forest and non-forest, and especially in cases where selective logging has resulted in small cleared areas in the forest matrix. The first two products, greenness and mid-infrared bands, help in differentiating forest from other types of vegetation, and the brightness bands in detecting non-forest areas. Training samples (i.e. 4,696 from 2005; 4,071 from 2000 3,447 from 1996, 2,748 from 1991; 3,481 from 1986) were digitized using the pre-classified image mosaics, recording known locations of forest, pasture and bare or built land cover. The latter two were combined into a non-forest class. The primary and secondary image products were used to get pixel values for these locations. Application of decision tree classification started with creating the decision rules with data mining software (Compumine, http://compumine.software.informer.com/), using 85% of the data to train and create the decision tree, and 15% to test the tree. To create classifications the ERDAS Knowledge Engineer rule-based classifier was used. Finally, more than 350 training sample points were collected in 2006 and 2007 with land cover type observations, used to evaluate accuracy of the 2005 classification. In addition, Advanced Spaceborne Thermal Emission and Reflection Radiometer (ASTER) images were used for an additional accuracy assessment of the 2000 and 2005 images with 600 points digitized across the region. Average accuracy of the classification for the 2005 images was 87.85% for the field samples, and 97.96% for the ASTER images (information on confusion matrices has been published in the previous study). Subsequently the classification method was applied to all images. With the community polygons, forest area (km^2^) and forest as proportion of the whole community area were calculated.

We also gathered data to measure the Enhanced Vegetation Index (EVI) and fire incidence. EVI is a reflectance-based index, essentially expressing “greenness” of an area^48,49^. We downloaded data from the Moderate Resolution Imaging Spectroradiometer (MODIS) from the Land Processes Distributed Active Archive Center (LP-DAAC, https://lpdaac.usgs.gov/data_access/data_pool). The product used was MOD13Q1, providing EVI at 250 m spatial resolution and essentially 16-day temporal resolution (the algorithm uses the best pixel value from a 16-day period). Valid values of EVI range between −0.2 and 1, with higher values reflecting higher levels of “greenness” (chlorophyll). Healthy vegetation generally falls between 0.20 and 0.80. EVI is sensitive to canopy structural changes and does not saturate as easily as other vegetation indices under high biomass conditions found in tropical areas. For data series per community and at a monthly time step, 16-daily data was averaged per month and GIS was used to extract area-weighted averages.

For fire time series, we drew on thermal anomalies in MOD14A2 data. These also come from MODIS, and were downloaded with a temporal resolution of 8 days and a spatial resolution of 1 km. In this product pixels have been assigned a classification of fire or no fire, creating a “fire mask”. For each community polygon, the proportion of pixels experiencing fire was calculated (for the 8-day data), which was then averaged over all fire masks in a month to obtain monthly values. The values thus represent the average area in a community that experienced fire in a particular month.

We also compiled Net Primary Productivity (NPP) time series (in kg C/m^2^) for the communities. NPP is available from the Numerical Terradynamics Simulation Group (NTSG) website (http://files.ntsg.umt.edu/data/NTSG_Products/MOD17/) and is the result of an algorithm that combines several factors. The Fraction of Photosynthetically Active Radiation (FPAR), Leaf Area Index (LAI) and land cover classification from MODIS are involved. Temperature, incoming solar radiation, and vapor pressure deficit from global reanalysis dataset are included, as well as a Biome Parameter Lookup Table with conversion efficiency parameters for different types of vegetation. Running and Zhao^50^ describe the algorithm that produces MOD17A3 (annual NPP at 1 km resolution) in detail, and Running *et al*.^51^ provide an in-depth explanation of the development of monthly data.

We obtained a longer time series for the Enhanced Vegetation Index (EVI2) from the University of Arizona’s Vegetation Index and Phenology lab (VIPLab, https://vip.arizona.edu/). They apply an algorithm to translate two-band data from the Advanced Very High Resolution Radiometer (AVHRR) into MODIS EVI (which uses 3 bands)^52^ to extend the EVI time series AVHRR back to 1982. The conversion method involves a calculation that expresses EVI2 as a function of the ratio of red to blue reflectivity. Before this conversion, the VIPLab applies a number of pre-processing steps, quality control, gap-filling, and calculations to ensure continuity across sensors. These processes are all outlined on the VIPLab website (https://vip.arizona.edu/viplab_data_explorer.php). The data were obtained in monthly time steps and at a 0.05° resolution for 1982–2010. Area-weighted time series were extracted for each community polygon. A number of corrections were applied to ensure continuity between pre- and post-2000 data (see “Technical Validation”)^53^. Note that there are differences between the EVI data described above and these EVI2 data for 2000–2010 due to the different resolutions of the data, and the modifications applied at the VIPLab.

**Fig. 2:**
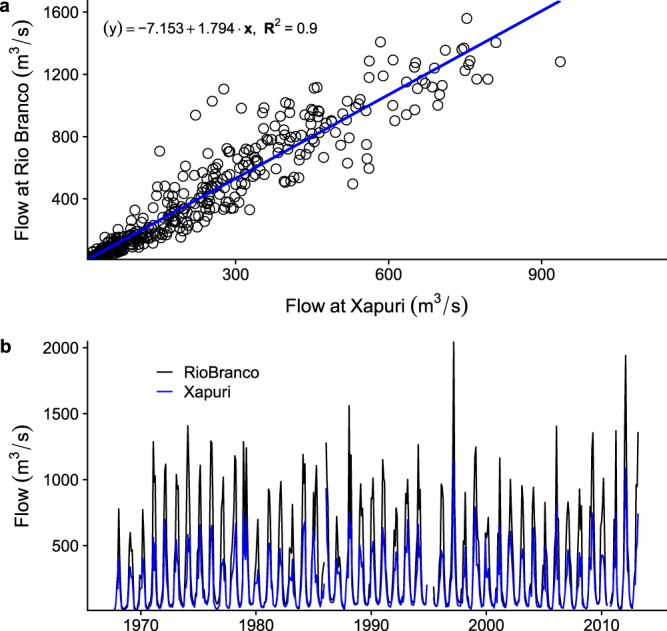
Stream flow data gap-filling information. (**a**) Relationship between hydrological stations at Rio Branco and Xapuri, used to gap-fill stream flow data. (**b**) Gap-filled stream flow data for Rio Branco and Xapuri (1967–2013).

Monthly precipitation data (in mm) were extracted from the Global Historical Climatology Network (GHCN) by NOAA (https://www.ncdc.noaa.gov/ghcnm/v2.php) for 4 stations in Acre (Brazil), 1 in Madre de Dios (Peru) and 2 in Pando (Bolivia). All these time series have gaps, ranging from a few months to a full year. Further, we downloaded monthly minimum, maximum and average stream flow data (in m^3^/s) for important rivers in the area. The data come from the Agência Nacional de Águas (ANA) in Brazil, through its Hidroweb (http://www.snirh.gov.br/hidroweb/ or http://hidroweb.ana.gov.br/). Searches were conducted to find stations for the two most important river basins: the Rio Acre, in sub-basin 13 of the Solimôes basin, and the Rio Madre de Dios, in sub-basin 15 of the Madeira basin. The Rio Acre forms part of the border between Peru and Brazil, and between Bolivia and Brazil (to Cojia/Brasiléia). It then turns north into Acre where it runs through the regional capital of Rio Branco. The Rio Madre de Dios originates in Peru, flows into Bolivia and eventually joins the Rio Madeira. Most stations have gaps in the data, and upstream/downstream station data and area-weighted precipitation data was explored for relationships to fill gaps. For 2 stations on the Rio Acre, Xapuri and Rio Branco, enough data was available to apply a gap-filling method for mean flow based on linear regression between station data to create a continuous time series (R^2^ = 0.90, see Fig. 2)^54^.

At the country level, we also compiled annual indicators from the World Bank Databank (http://databank.worldbank.org/data/home.aspx): agricultural land, arable land and protected areas, all as percentage of the land area in each country.

At the global level, we obtained monthly time series for the Atlantic Multidecadal Oscillation (AMO), the Pacific Decadal Oscillation (PDO) and the Multivariate El Niño/Southern Oscillation Index (MEI) from the NOAA Earth System Research Laboratory (https://www.esrl.noaa.gov/psd/data/climateindices/list/). These time series relate to sea surface temperatures and can often be related to global, hemispherical and continental carbon fluxes and climatic variables^55^, making them valuable indicators and predictors of change. For the AMO, we obtained the unsmoothed version^56^: this time series is an index of surface temperature of the North Atlantic Ocean. The PDO consists of the first principal component of monthly anomalies of sea surface temperature of the North Pacific Ocean^57,58^. The MEI is a composite index that is believed to better reflect the El Niño/Southern Oscillation phenomenon than simply sea surface temperatures^59^. MEI incorporates sea level pressure, surface air temperature, sea surface temperature, cloudiness fraction, zonal and meridional components of surface wind over the tropical Pacific Ocean.

### Stationary variables

We also include stationary data for the communities in this database. These data are not necessarily data that never change, but rather data for which there are currently only “snapshots” available, not time series. These records contain information on names, land areas, distances to markets and capitals (see “Human variables”) and mean elevation (meters above sea level, MASL). Mean elevation is extracted from a Digital Elevation Map (DEM) using the “Zonal Statistics” tool in ArcGIS. This DEM was sourced from the website of the United States Geological Society (USGS, http://gdex.cr.usgs.gov/gdex/) associated with LP-DAAC. The community records contain community IDs to link the various files with community level data. The results from the household surveys, mentioned in the section “Human Variables”, are also included as separate files for the three states, Acre, Madre de Dios and Pando. The survey questionnaire asked about household location, migration history, road access, household assets (human, physical and social capital), land tenure, livelihood activities, health and well-being, past changes and future plans. Questions on livelihood activities also enquired about resource management, such as agriculture, livestock and forest extractive activities.

The forest type data were created using a random forest^60^ (RF) model and a simplified classification from Salimon *et al*.^61^ for Acre, Brazil and applied to Pando, Bolivia and Madre de Dios^62^. We created training samples for five dominant forest types (Dense, Palm, Bamboo, Alluvial and Sub-montane) with spatially limited classes of grasslands, wetland and sub-montane. We inserted bamboo data based on 2001–2008 presence data from de Carvalho *et al*.^31^. We created covariates from the WorldClim Bioclim data^63^, SoilGrids1km^64^, and MODIS MOD13A3 1km 16-day composite EVI^48^ from 2000 to 2014. We condensed the MOD13A3 EVI into annual average composites, and monthly average composites across all years. We also calculated annual and monthly averaged composites for 2006–2011, the years matching our remote sensing and botanical field sampling campaigns.

**Fig. 3:**
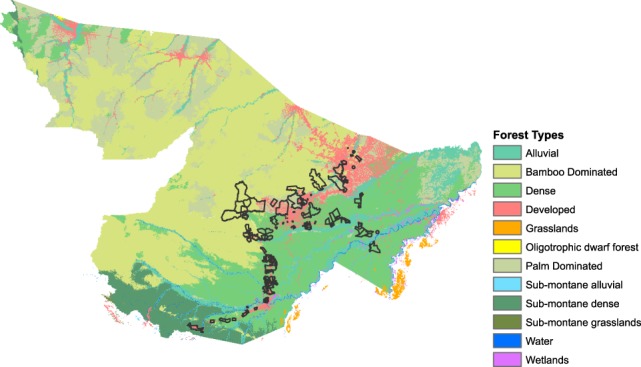
Forest types generated from the Random Forest (RF) model, with community polygons.

Using the randomForest package^65^ from R we fit a full RF model with an initial forest size of 500 trees and an initial node size of one, later changed to three. Non-forest pixels were removed from the estimation process. We optimized the model by iteratively removing any covariates with negative increases in node purity indicated by an increase of Mean Decrease Accuracy, a measure of how much the inclusion of a predictor in the model reduces out-of-bag (OOB) error^60^. This process began with a list of covariates with an importance score greater than zero. We selected the top ten variables, based on mean decreased accuracy identified in variable importance plot, to create final prediction of forest type, Fig. 3.

Botanical data was collected in the area between 2007 and 2009 for analysis of tree species diversity and composition, aboveground biomass^66^ and forest value^38^. Because published articles include in-depth discussions of the field and laboratory methods employed, here we provide a summary. Study sites were selected to encompass geographic variability, and community leaders were engaged to provide consent and identify representative areas in their forests. We focused on terra firme forests with the aim of being able to evaluate human impacts. Across the MAP frontier, we sampled 67 sites using an adaptation of the Phillips *et al*.^67^ modified Gentry plot method^68^ with paired cases near and far from the IOH where possible, in the communities selected. There were 27 sites in Acre, 25 in Madre de Dios and 15 in Pando. Distance from roads varied, with generally one site closer to roads (<2 km) and 1 further away (>5 km) in each community. Each site consisted of a 100 × 190 m sampling grid, within which 10 subplots of 2 × 50 m were sampled. The subplots were situated perpendicular to a baseline that was established to represent a homogeneous and representative area of the surrounding forest, in terms of composition and impacts of any disturbances. Woody plants with a diameter at breast height (DBH, at 1.3 m) ≥2.5 cm were measured for height and DBH and identified to species in the field, or an herbarium voucher was taken. To improve the precision of estimates of aboveground biomass and forest structure in most plots, each subplot was extended to 10 × 50 m, and all woody stems with DBH ≥20 cm were measured and identified rapidly to as high a taxonomic level as possible. Voucher specimens were collected for species that could not be identified in the field, and collections from each country were deposited at herbaria in regional universities: the National Amazonian University of Madre de Dios in Puerto Maldonado (Peru), the Center for Research on Amazon Protection of the Amazonian University of Pando in Cobija (Bolivia), and the Zoobotanical Park of the Federal University of Acre in Rio Branco (Brazil). After extensive identification efforts for the 0.1 ha floristic plots, 93.9% of samples were identified to at least the genus level; among the additional larger stems inventoried for biomass estimations, 88% of stems were identified to at least the genus level. In addition to detailed plant diversity and composition data, aboveground biomass was estimated with allometric equations based on empirical diameter and height data and wood density assigned based on genus identity, as per Baraloto *et al*.^68^.

To obtain soil information for the area, we downloaded a gridded soil dataset from the International Soil Reference and Information Centre (ISRIC – World Soil Information, http://www.isric.org/explore/soilgrids). This dataset has a resolution of 1 km and provides soil organic carbon (g/kg), soil pH, sand, silt and clay fractions (%), bulk density (tonnes/m^3^), cation-exchange capacity (cmol/kg), volumetric coarse fragments, soil organic carbon stock (tonnes/ha) at 6 depths covering 0–200 cm soil profiles, and depth to bedrock (m)^64^. For this database, we depth averaged the six values to obtain single values. Values for saturated hydraulic conductivity (10^−6^ m/s), saturated volumetric water content and residual volumetric water content (both cm^3^/cm^3^) were calculated with pedotransfer functions listed in Table 2 in Marthews *et al*.^69^, using those by Cosby *et al*.^70^ for saturated hydraulic conductivity and those by Tomasella and Hodnett (1998)^71^ for water content. We extracted information for each community using GIS.

We also included shapefiles for state boundaries, communities, towns, hydrological and meteorological stations, roads, rivers, protected areas and reserves, elevation and soil information. The metadata for the point data (flow and precipitation from stations) provide coordinates for the stations. The elevation raster was sourced from the U.S. Geological Survey (USGS) website (http://gdex.cr.usgs.gov/gdex/), and the soil raster from the aforementioned ISRIC source.

## Data Records

All datasets and records are stored in an *Open Science Framework* repository^72^. The main level of data organization is under the headers “human variables”, “natural variables” and “stationary data” (Online-only Tables 1 and 2). Within these, there are subdivisions. Under “human variables”, data are grouped at either the community or country level. Online-only Table 1 lists the available data and their level, and files are named according to the listed abbreviation. All data are provided in comma-delimited files (.csv), with either a country or community identifier. Where necessary, metadata files are included to elaborate on data in the files. Natural variables have a similar format, and also contain data at global or point level. Data at global level (climate indices) are not linked to any country or community. Point data files (meteorological and hydrological data) contain coordinates for station locations in a metadata file, including station numbers. Separate files contain the flow data per station, since these data contain several time series. For precipitation, data are all contained in one file. These files are also in comma-delimited format. The temporal resolution of the dynamic variables is indicated in Online-only Table 1. Data records contain columns indicating months and years for ease of reading dates for time series analysis. The time periods for which data is available varies; see Online-only Table 1.

Stationary data are a mix of comma-delimited files, shapefiles (.shp and associated files) and rasters (.tif and.adf). Data are grouped by community information, botanical data, forest type, shapefiles and soil type (Online-only Table 2). Shapefile information, such as projections and attributes, are contained in a README file. All other files are comma-delimited and are accompanied by a metadata file.

Three *figshare* archives contain details on calculations of the EVI2 time series^53^, gap filling of flow time series^54^ and the forest type classification and mapping^62^.

## Technical Validation

Data from reanalysis projects or large global datasets were sourced from reputable sources (e.g. WorldBank data, data from NOAA, CRU, ISRIC) with internal data checks. Local data were obtained from sources with documentation on methods of measurement and calculations (e.g. ANA, GHCN). Visual checks of data relating to dynamic variables were done to ensure there were no anomalous data. Where there were minor issues with community data relating to human variables (e.g. travel times, family density), these were identified and resolved in an iterative manner, with several researchers evaluating the datasets. Note that some station data (precipitation and stream flow) have gaps.

**Fig. 4:**
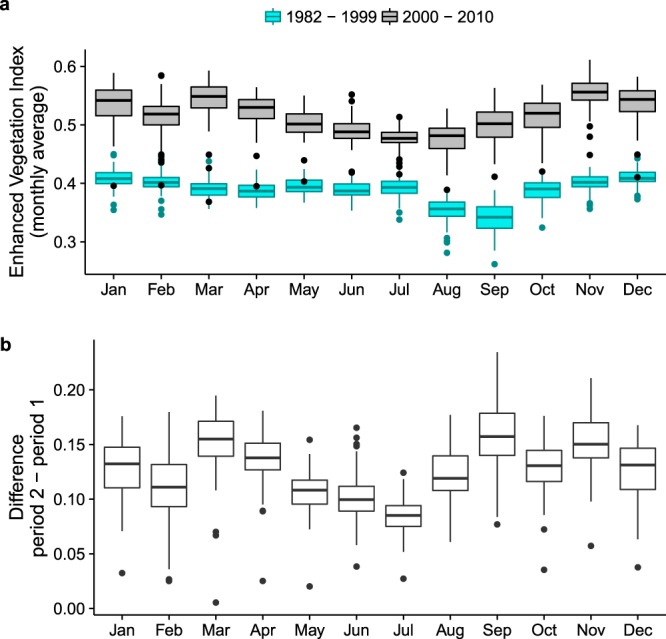
Information used in technical validation and correction of EVI2 time series per community. (**a**) Average monthly EVI2 for 2 periods, 1982–1999 (AVHRR-derived) and 2000–2010 (MODIS). Boxplots indicate the distribution of values across 100 communities. (**b**) Differences in average EVI2 values between the 2 periods. Boxplots indicate the distribution of values across 100 communities.

Data correction was performed on EVI2 data to address outliers and discrepancies between AVHRR-derived EVI (1982–1999) and MODIS EVI (2000–2010), because AVHRR-derived EVI exhibited consistently lower values (Fig. 4a). This is attributed to the lower quality of AVHRR data in areas with high cloud density (pers. comm. Dr. K. Didan). For this correction, we divided data into pre- and post-2000 data. For each, we identified outliers as uncharacteristically positive changes from one month to another. Negative changes were not scrutinized, since vegetation can be cut or burned down, causing large negative change in EVI. However, it would be unlikely for EVI to suddenly increase. Any change larger than 2 times the interquartile range of the data was flagged and removed. The change value was purposefully kept large since the data had already undergone pre-processing at the VIPLab. Removed values were replaced with long-term averages on a month-by-month basis (e.g. a gap for January was filled with the long-term average for January). The pre-2000 dataset was also adjusted by moving the data up by the value of the long-term post-2000 average, again on a month-by-month basis for each community. This was done on a month-by-month basis because differences between the long-term averages varied per month (Fig. 4b). An RMarkdown file with code and this explanation is available from a *figshare* repository^53^.

The RF classifier of forest types provide an internal validation measure and assessment of individual contribution from covariates. The out-of-bag (OOB) error is calculated from withheld data from the training data set. It is used as internal validation during the estimation step. The initial OOB error for the forest type classification was 12.6%. The Mean Decrease in Accuracy was calculated during the OOB error calculation part of estimation process. We then ran an interactive classification to remove covariates that contributed negative increases in node purity to remove computation burden during the prediction stage. An internal 10-fold cross-validation of the training set was calculated during the final, iterative classification which produced a final classification accuracy of 94%.

Botanical data reflect updates for herbarium vouchers that were completed in 2013 and 2014 in the regional herbaria where limited reference vouchers remain available. Taxonomic hypotheses have been checked and updated for synonymy using the Taxonomic Name Resolution Service (TNRS) application^73^. At the time of this publication, only 41.6% of stems have been accurately identified to the species level, even though 93.9% of stems have been identified to the genus level.

We highly encourage users to consult the original sources, websites and publications for general data limitations and caveats. While all reanalysis and calculated data comes from reputable sources, underpinned by peer-reviewed publications, users should familiarize themselves with metadata and take processing and correction methods into account. All variables have been measured in the International System of Units (SI). Note that Portuguese or Spanish names can include special characters that may not transfer into coding or analysis applications.

## Usage Notes

**Fig. 5:**
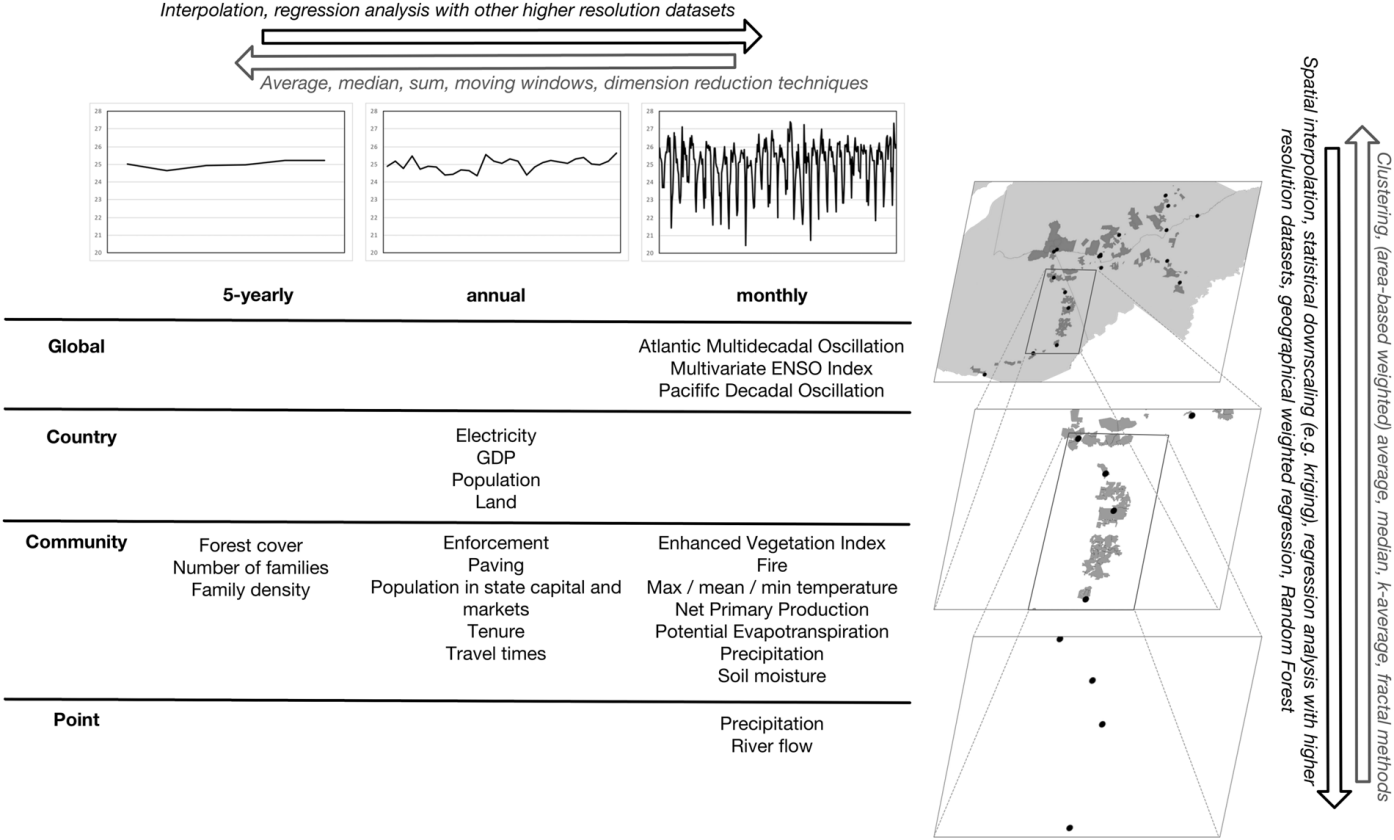
Schematic and tabular overview of spatial and temporal dimensions and examples of up- and downscaling techniques.

This dataset covers several spatial and temporal scales, Fig. 5 provides a schematic summary with examples of methods that can be employed to match scales. The time spans covered are listed in Online-only Table 1, the spatial area covered for the states ranges from -73.989707 to -65.27995 latitude and -13.36179 to -7.12132 longitude (WGS 1984 projection). For matching the various data types, the file “Communities.csv” in the folder “Community_info” in “Stationary data” is key. This file contains the name and country of each community, including an ID number (e.g. X_001, X_002). In turn, each data type has a reference to either country or community ID (e.g. FT_001 for the forest type in community X_001, MINT_007 for minimum temperature in community X_007, GDP_PER for GDP for Peru). The botanical data is linked to countries and has coordinates. Flow and precipitation data at station level have country names, station numbers and coordinates in the metadata file. The household survey data is more complicated, with communities referenced by name and countries by numbers. We refer to the metadata file for details on matching households, communities and countries.

As the impact of human disturbances increases in many areas due to infrastructure development, it is important to be able to analyze systems as broadly as possible. Considering affected regions as coupled human and natural systems that are dynamic over time and space allows researchers to fully explore different types of impacts. We encourage researchers to use our data in explicitly spatiotemporal approaches integrating human and natural variables. While previous studies have already been conducted with our data, each featured specific components of the broader dataset. Of particular interest for future research would be to analyze changes over time in the system, but these data could also be used to investigate spatial variability and heterogeneity as a consequence of road infrastructure. With the available data, analyses can take place at regional or community levels. Of interest could be:

1. Time series research on dynamic variables to understand the interplay between drivers, response variables and their feedbacks;
2. Simulation and prediction studies to identify important variables for system state changes. This type of research would be informative for other regions undergoing road infrastructure upgrades; and
3. Global Sensitivity and Uncertainty Analyses (GSUA) to evaluate the workings of simulation and prediction models. The data in this database can provide a baseline for the development of model input probability distributions required for GSUA.

While our dataset is comprehensive, we acknowledge there are other projects and datasets that are also useful for analysis of coupled human and natural systems. Our dataset was compiled to emphasize data available uniformly over time and space across the study region. Consequently, governmental data without annual time series (such as national census data) were not included. We nonetheless encourage researchers to use other data sources in conjunction with our dataset, especially when focusing on one country within our study area. Examples would be Brazilian social indicators data (which are often more comprehensive than for the other countries), deforestation monitoring initiatives such as the Amazon Deforestation Program (PRODES) of Brazil’s Ministry for Science and Technology (MCT), or data from the Large Scale Biosphere-Atmosphere Experiment (LBA-ECO) implemented by the US National Aeronautics and Space Administration (NASA) and Brazil’s MCT.

### ISA-Tab metadata file

Download metadata file

## Data Availability

The code written and used for the development of the forest type, EVI2 and river flow data is available in the repositories specified in the references^53,54,62^. Programs used, including versions, are outlined in the repository descriptions.

## Online-only Table

**Online-only Table 1:** Overview of dynamic variables collected and compiled for the area in Madre de Dios (Peru), Acre (Brazil) and Pando (Bolivia), the MAP area.

Sub-system	Spatial resolution	File name (and abbreviation)	Dynamic variables (units)	Temporal resolution	Time period
HUMAN	Community	Enforcement (ENF)	Enforcement of tenure rules (0 to 1: with 0 = least, 1 = most)	Annual	1985–2010
	Community	Families (FAM)	Number of families in a community	5-yearly	1987–2012
	Community	Family density (FAMD)	Family density (families/km^2^)	5-yearly	1987–2012
	Community	Paving (PAV)	Paving (0 to 1, with 0 = no paving, 1 = fully paved)	Annual	1986–2010
	Community	PopulationCapital (PC)	Population in the state capital	Annual	1986–2010
	Community	PopulationMarket (PNM)	Population in nearest market	Annual	1986–2010
	Community	Tenure (TEN)	Percentage of deforestation allowed under tenure rules (0 to 1: e.g. 0.1 = maximum of 10% deforestation allowed)	Annual	1985–2010
	Community	TraveltimeCapital (TTC)	Travel time to capital (minutes)	Annual	1985–2012
	Community	TraveltimeMarket (TTM)	Travel time to nearest market (minutes)	Annual	1985–2012
	Country	Electricity (ELNR)	Electricity from non-renewable sources (oil, gas and coal; % of total)	Annual	1980–2010
	Country	Electricity (ELPC)	Power consumption (kWh per capita)	Annual	1980–2010
	Country	Electricity (ELREN)	Electricity from renewable sources, excluding hydroelectric (% of total)	Annual	1980–2010
	Country	GDP (GDP)	GDP, PPP (current international $) = GDP converted to international dollars using purchasing power parity rates	Annual	1980–2010
	Country	GDP (GDPFOR)	Profit from forests (% of GDP)^a^	Annual	1980–2010
	Country	GDP (GDPGAS)	Profit from natural gas (% of GDP)^c^	Annual	1980–2010
	Country	GDP (GDPGROW)	GDP growth (annual %)	Annual	1980–2010
	Country	GDP (GDPGROWCAP)	GDP growth per capita (annual %)^b^	Annual	1980–2010
	Country	GDP (GDPINV)	Foreign direct investment, net inflows (% of GDP)	Annual	1980–2010
	Country	GDP (GDPMIN)	Profit from minerals (% of GDP)^d^	Annual	1980–2010
	Country	GDP (GDPNAT)	Total profit from natural resources (% of GDP)^e^	Annual	1980–2010
	Country	GDP (GDPOIL)	Profit from crude oil (% of GDP)^f^	Annual	1980–2010
	Country	Population (POPLIFE)	Life expectancy at birth, total (years)	Annual	1980–2010
NATURAL	Point	Hydro_stations (FLOW)	River flow at a number of stations	Monthly*	~1967–2013*
	Point	Meteo_stations (PP)	Precipitation (mm) at a number of meteorological stations	Monthly*	~1985–2011*
	Community	EVI (EVI)	Enhanced Vegetation Index (MODIS product)	Monthly	2000–2010
	Community	EVI2 (EVI2)	Enhanced Vegetation Index 2 (VIPLab product)	Monthly	1982–2010
	Community	Fire (FIR)	Fire occurrence (average over a community area per month)	Monthly	2000–2010
	Community	ForestCover (FOR)	Forest area as percentage of community area	5-yearly	1986–2010
	Community	InferredSpeciesRichness (SR)	Inferred species richness (alpha diversity)^g^	Annual	1984–2012
	Community	MaxTemp (MAXT)	Maximum temperature (°C)	Monthly	1982–2010
	Community	MeanTemp (MEANT)	Mean temperature (°C)	Monthly	1982–2010
	Community	MinTemp (MINT)	Minimum temperature (°C)	Monthly	1982–2010
	Community	NPP (NPP)	Net Primary Production (C/m^2^)	Monthly	2000–2010
	Community	PET (PET)	Potential evapotranspiration (mm)	Monthly	1982–2010
	Community	Precipitation (P)	Precipitation (mm)	Monthly	1982–2010
	Community	SoilMoisture (SM)	Soil moisture (mm)	Monthly	1982–2010
	Country	Land (LANDAG)	Agricultural land (% of land area)^h^	Annual	1980–2009
	Country	Land (LANDAR)	Arable land (% of land area)^i^	Annual	1980–2009
	Country	Land (LANDPROT)	Percentage Protected Areas (% of whole country)	Annual	1990–2010
	Global	PDO_MEI_AMO (AMO)	Atlantic Multidecadal Oscillation (unsmoothed and smoothed)	Monthly	1987–2015 (unsmoothed) 1980–2012 (smoothed)
	Global	PDO_MEI_AMO (MEI)	Multivariate ENSO Index	Monthly	1980–2015
	Global	PDO_MEI_AMO (PDO)	Pacific Decadal Oscillation	Monthly	1980–2015

^a^Also called ‘forest rents’, defined as the roundwood harvest multiplied with the product of average prices and a region-specific rental rate.

^b^GDP per capita based on purchasing power parity (PPP). PPP GDP is gross domestic product converted to international dollars using purchasing power parity rates. An international dollar has the same purchasing power over GDP as the U.S. dollar has in the United States. GDP at purchaser’s prices is the sum of gross value added by all resident producers in the economy plus any product taxes and minus any subsidies not included in the value of the products. It is calculated without making deductions for depreciation of fabricated assets or for depletion and degradation of natural resources. Data are in current international dollars.

^c^Also called ‘natural gas rents’: the difference between the value of natural gas production at world prices and total costs of production.

^d^Also called ‘mineral rents’: the difference between the value of production for a stock of minerals at world prices and their total costs of production. Tin, gold, lead, zinc, iron, copper, nickel, silver, bauxite and phosphate are included.

^e^Also referred to as ‘total natural resources rents’: the sum of oil rents, natural gas rents, coal rents, mineral rents and forest rents.

^f^‘Oil rents’: the difference between the value of crude oil production at world prices and total cost of production.

^g^Alpha diversity translated into species richness per community, inferred from Landsat data by Convertino *et al*.^41,42^.

^h^Agricultural land refers to the share of land area that is arable, under permanent crops, and under permanent pastures. Land under permanent crops is land cultivated with crops that occupy the land for long periods and need not be replanted after each harvest, such as cocoa, coffee, and rubber. This category includes land under flowering shrubs, fruit trees, nut trees, and vines, but excludes land under trees grown for wood or timber. Permanent pasture is land used for five or more years for forage, including natural and cultivated crops.

^i^Arable land includes land defined by the FAO as land under temporary crops (double-cropped areas are counted once), temporary meadows for mowing or for pasture, land under market or kitchen gardens, and land temporarily fallow. Land abandoned as a result of shifting cultivation is excluded.

*Varying start and end dates. Some time series have gaps, for flow 2 stations have been gap-filled (see ‘Methods’). Time series vary in length for each station.

**Online-only Table 2:** Overview of stationary (snapshot) data collected and compiled for the area in Madre de Dios (Peru), Acre (Brazil) and Pando (Bolivia), the MAP area.

Sub-system	Spatial resolution	Stationary (snapshot) variables	
HUMAN	Community	Community characteristics	Community ID Community names Coordinates Community area (km^2^) Country Area as fraction of study area Area as fraction of study area in each country Elevation, mean and standard deviation (MASL) Capital Distance to (state) capital (km) Nearest market Distance to nearest market (km)
	Households	Household characteristics	Various, themes: Location - Migration history - Household assets • Human capital • Housing quality and durable goods • Durable goods for transport, communication • Social capital • Capital inputs for household productive activities - Land tenure - Productive activities • Agriculture • Forest extractivism 2005–2008 - Health and well-being (last 12 months) - Past changes (last 5 years) and future plans (next year)
NATURAL	Community	Soil types	Soil organic carbon (g/kg) Soil pH Sand, silt and clay fractions (%) Bulk density (tones/m^3^) Cation-exchange capacity (cmol/kg) Volumetric coarse fragments Soil organic carbon stock (tonnes/ha) Depth to bedrock (m) Saturated hydraulic conductivity (10^−6^m/s) Saturated volumetric water content (cm^3^/cm^3^) Residual volumetric water content (cm^3^/cm^3^)
	Community	Forest types	Alluvial (%) Bamboo dominated (%) Palm dominated (%) Dense (%) Developed (%) Water (%) Wetlands (%) Submontane dense (%)
	Transects	Botanical data	Per transect: - Family - Genus - Species - Diameter at breast height (cm) - Height (m) - Voucher Per plot: - Year - Distance from Inter Oceanic Highway (m) - Distance from nearest urban area with population > 25000 (m) - Distance from nearest point in Andean area with MASL > 1000 m - Aboveground biomass (Mg/ha)
GENERAL	Variable	Shapefiles	State boundaries Community boundaries Protected areas and reserves Roads Rivers Soils Digital Elevation Map Stations (hydrological and meteorological) and towns
